# Analysis of mitochondrial DNA sequence and copy number variation across five high-altitude species and their low-altitude relatives

**DOI:** 10.1080/23802359.2018.1501285

**Published:** 2018-08-27

**Authors:** Rui Liu, Long Jin, Keren Long, Qianzi Tang, Jideng Ma, Xun Wang, Li Zhu, An’an Jiang, Guoqing Tang, Yanzhi Jiang, Xuewei Li, Mingzhou Li

**Affiliations:** Farm Animal Genetic Resources Exploration and Innovation Key Laboratory of Sichuan Province, Sichuan Agricultural University, Chengdu, China

**Keywords:** High-altitude adaption, mitochondrial DNA, mtDNA copy number, high- and low-altitude species

## Abstract

High-altitude inhospitable environments impose a formidable life challenge for the local animals. Training and exposure to high-altitude environments produce both distinct physiological and phenotypic characteristics. The mitochondrion, an organelle crucial for the energy production, plays an important role in hypoxia adaptation. In this study, we investigated the mitochondrial DNA (mtDNA) polymorphism and copy number variation between the population pairs from distinct altitudes across the multi-species. Higher mitochondrial DNA control region’s genetic diversity is conspicuous in high-altitude animals versus low-altitude relatives. We also found an accordant decrease of mtDNA copy number in most of the tissues from high-altitude animals. Compared to mammals, chickens have significantly distinct mitogenomic characteristics, and more significant changes in the skeletal muscle mtDNA copy number between high- and low-altitude individuals. Our study catches a snapshot of the biological similarities and differences in the mitochondrial high-altitude acclimation across the species.

## Introduction

Hypobaric hypoxia is associated with the extreme environmental characteristics of high-altitude environments, which is much more than that of lowland environments at similar latitudes. This imposes strong selective pressure on high-altitude animal species, both native and migratory. Compared with low-altitude animals, these animals possess unique high-altitude physiological adaptations and evolutionarily derived complex genetic architectures (Grether 2005). Tremendous genomic analyses have identified a vast array of rapidly evolving genes, contributing to the phenotypic change in the high-altitude animals (Simonson et al. 2010; Qiu et al. 2012; Li et al. 2013; Lorenzo et al. 2014). Recent comparative genomic analyses have revealed convergence and divergence in the gene expression changes between the species while adapting to high-altitude environments (Tang et al. 2015; Tang et al. 2017). Mitochondria, having an indispensable role in the energy metabolism, being extremely sensitive to the different energy-related selective pressures, more than the nuclear genome, is a core feature of extreme high-altitude acclimatization (Shen et al. 2009; Murray and Horscroft 2016). Mitochondrial genetic and phenotypic variations for high-altitude adaptation have been reported in mammals, including, among others, the Tibetan yak, Tibetan sheep, Tibetan pig, and bar-headed goose (Scott et al. 2011; Gu et al. 2012; Nabholz et al. 2013; Hodgkinson et al. 2014; Li et al. 2016; Liu et al. 2016). In this study, focus had been laid on the mitochondrial genome and mitochondrial copy number in the population pairs from distinct altitudes within a particular species. An attempt has been made to catch a glimpse of the consistencies and differences in the mitochondrial signatures shaped by high-altitude adaptation across multi-species.

## Materials and methods

**Figure 1.**
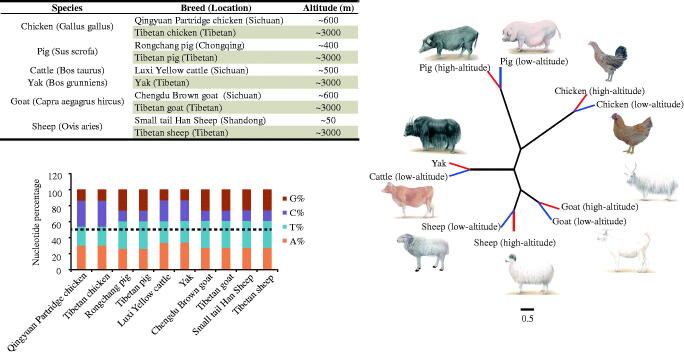
Characteristics and phylogenetic analysis of mitogenome in five high-altitude species and their low-altitude relatives. (a) Detailed information of animals and geographical locations. b. Base content of mitochondrial genome across five species. c. Neighbor joining tree of five species, based on complete mtDNA sequences (Accession numbers: MG837547-MG837556); red branches denote high-altitude animals, and blue branches denote low-altitude animals.

The detailed information of the animals and geographical locations are shown in Figure 1(a). The liver, brain, heart, lung, and skeletal muscle (the *longissimus dorsi* in four mammals and the chest muscle in chicken) were collected and used to extract complete DNA using MicroElute Genomic DNA kit (Omega, GA). Mitochondrial DNA (mtDNA) sequences from ten breeds were sequenced by an ABI 3730 DNA sequencer (Applied Biosystems, Foster City, CA). The mtDNA sequences in this study (GenBank accession numbers: MG837547-MG837556) were assembled manually using the computer program DNASTAR. A neighbor-joining tree was constructed using the Kimura 2-parameter method with software MEGA 6.0 (Tamura et al. 2013). Sequences of mitochondrial DNA D-loop region were downloaded from the GenBank (Table 1) and were used to calculate the nucleotide diversity (*π*), using the DNAsp software (Librado and Rozas 2009).

**Table 1.** Genetic diversity analysis of the mtDNA D-loop region between high-altitude (from Tibet) and low-altitude animals (mostly from Sichuan basin).

Species	Population (Location)	*n*	*π* ± *s*	GenBank accession numbers
Chicken	Indigenous chicken (Sichuan)	5	0.00267 ± 0.00061	GU448683.1-GU448687.1
	Tibetan chicken (Tibetan)	23	0.01250 ± 0.00204	GU448036.1-GU448050.1, GU448893.1-GU448900.1
Pig	Rongchang pig (Chongqing)	31	0.00394 ± 0.00035	JX068465.1-JX068474.1, JX068391.1-JX068400.1, JX068439.1, JX068495.1-JX068504.1
	Tibetan pig (Tibetan)	33	0.00509 ± 0.00051	JX068268.1-JX068275.1, JX068286.1-JX068293.1, JX068344.1-JX068353.1,JX068365.1-JX068371.1
Cattle	Indigenous cattle (Sichuan)	23	0.05216 ± 0.00971	AY902385.1-AY902397.1, AY521083.1-AY521093.1, AY521112.1-AY378119.1
	Yak (Tibetan)	23	0.81506 ± 0.08030	MG213690.1-MG213712.1
Goat	Indigenous goat (Sichuan)	24	0.02469 ± 0.00440	JN828578.1-JN828598.1, JN828599.1-JN828607.1
	Tibetan goat (Tibetan)	36	0.02740 ± 0.00327	KY112615.1-KY112617.1, KY112622.1-KY112623.1, KY112628.1-KY112633.1, KY112655.1-KY112657.1, KY112664.1-KY112667.1, KY112675.1-KY112685.1, KY112702.1-JN828585.1
Sheep	Small tail Han Sheep (Shandong)	14	0.65997 ± 0.09824	FJ545863.1-FJ545876.1
	Tibetan sheep (Tibetan)	25	0.69226 ± 0.07614	AY829416.1-AY829422.1, DQ903190.1-DQ903206.1

*π:* denotes nucleotide diversity; *s:* denotes standard deviation.

Relative amounts of nuclear DNA and mtDNA were determined using quantitative RT-PCR as previously described (Miller et al. 2003). The mitochondrial genes *ATP6*, *COX1*, and *ND1* were used to quantify mtDNA. The *GCG*, *AGRT1*, *SCD*, *CSN2β*, and *AGRT1*, was used as the single-copy nuclear gene for pig, cattle/yak, sheep, goat, and chicken, respectively. Twice the ratio of *ATP6*/*COX1*/*ND1* to single-copy nuclear gene number reflected the relative mtDNA content. Primer sequences used for mtDNA sequencing and mtDNA copy number detection are provided in Supplementary materials (available at https://doi.org/10.6084/m9.figshare.6603149).

## Results and discussion

### Characteristics and phylogenetic analysis of mitogenomes

The mitochondrial genome sequences of ten breeds, within five species (pig, cattle, sheep, goat, and chicken, from high- versus low-altitude regimes) were determined. Mitogenomes were all ∼16.5 kb in length with heavy nucleotide biases toward A and T (accounting for 60%), except in chicken (Figure 1(b)), which showed a relatively balanced nucleotide composition. Additionally, based on phylogenetic analysis, mammals sort into bird (chicken), omnivore (pig), and ruminant (goat, sheep, and cattle/yak) clades (Figure 1(c)), agreeing with established phylogenetic relationships across these species (Nabholz et al. 2009; Nabholz et al. 2013).

### Nucleotide diversity of the mtDNA D-loop region

Mitochondrial DNA genetic markers can partly reflect the population fluctuations associated with local adaptations, especially the relatively sensitive mitochondrial control region (D-loop region) (Hassanin et al. 2009; Cheng et al. 2011). Here, we determined the similarities and differences in nucleotide diversity within the mtDNA D-loop region in a large-scale population analysis between high- and low-altitude species (Table 1). We observed relatively higher genetic diversity in the mtDNA D-loop region of high-altitude animals, which corresponds to previous results in the Tibetan pig (Li M et al. 2016), Tibetan sheep (Niu et al. 2017), Tibetan goat (Deng et al. 2017), and yak (Yue et al. 2016). We attribute the phenomenon to geographically isolating environments, and different domestication histories between the high- and low-altitude animals. Stronger artificial selection in low-altitude populations may contribute to the genetic diversity attenuation. Additionally, we found that different species exhibit different fold changes in the genetic diversity between high- and low-altitude populations, ranging from 1.05 in sheep to 15.6 in cattle/yak. The high-level diversity change in cattle/yak may be attributed to their highly diverged relationship (split from each other before 1.05–1.50 million years ago) (Qiu et al. 2012; Bai 2015). Interestingly, we found a significantly greater change in the genetic diversity (4.68 fold change in chicken, compared with 1.29 in pig, 1.11 in goat, 1.05 in sheep) between the pairs compared in bird than so in mammal.

### Detection of mtDNA copy number

**Figure 2.**
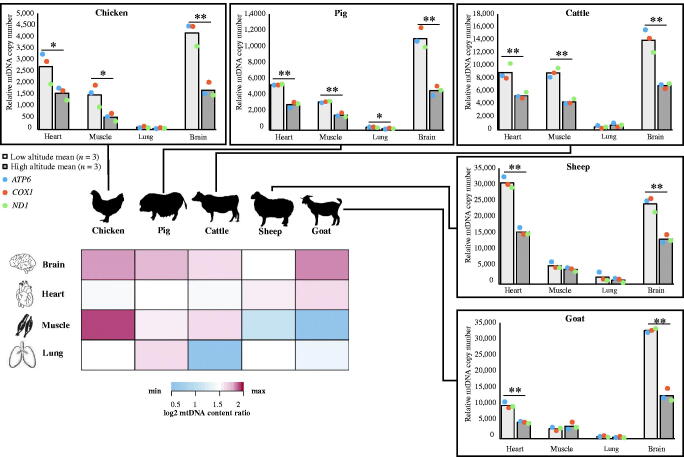
mtDNA copy number analysis of four tissues across five species, and a heatmap of mtDNA copy number ratio between low- and high-altitude animals (*n* = 3/group) in those four tissues. Student’s *t*-test, *p < 0.05, **p < 0.01. Colored entries represent the log_2_ mtDNA copy number ratio between low-altitude and high-altitude animals. The significant value was regarded as 1.5.

The decrease in mtDNA copy number associated with local animals living on the Tibetan Plateau has been demonstrated to be a long-term adaptive change contributed to high-altitude hypoxia adaption. We first investigated the mtDNA copy number in the population pairs from distinct altitudes within each group of a specific tissue (heart, skeletal muscle, lung, and brain) (Figure 2). For most of these tissues, we found the mtDNA copy number of high-altitude animals were lower than low-altitude counterparts. The result is consistent with the previous observations of decreased mitochondrial content with a concurrent smaller production of reactive oxygen species (ROS) across the vertebrate animal tissues, both *in vitro* and *in vivo*, when exposed to chronic hypoxia (Zhang et al. 2008; Heather et al. 2012; Li Y et al. 2016; Niu et al. 2017; Jin et al. 2018). However, increase in mitochondrial density occur in the early phases of acclimatization in response to acute hypoxia, which increases the metabolic cost of oxygen at the expense of cell damage by harmful ROS (Luo et al. 2011; McElroy and Chandel 2017). Moreover, we found the mtDNA copy number in highly aerobic brain (Larson et al. 2014) and heart tissues (Nakada et al. 2017) to exhibit higher copy number levels than in other tissues, meanwhile exhibiting a relatively greater change ratio of mtDNA copy number between low- and high-altitude animals across five species (Figure 2). The lung, as the primary respiratory organ, exhibited relatively blunting response to the altitude in mitochondrial content change across the five species, and the lowest copy number levels of the four tissues. Interestingly, we observed the greatest mtDNA copy number change in chicken skeletal muscle (Figure 2). This observation raises the point that birds might have distinct mitochondrial phenotypic acclimatization processes for high-altitude hypoxia compared with mammals. Consistent with this, a previous study found hundreds of genes related to aerobic metabolism were differentially expressed in skeletal muscle between high- and low-altitude sparrows (Cheviron et al. 2008). Other studies have demonstrated different mtDNA sequence features (Nabholz et al. 2009) and physiological adaptations to high altitudes (Scott et al. 2009; Scott 2011; Murray and Horscroft 2016) between birds and mammals.

## Conclusions

In this study, we determined pairwise differences in mitochondrial DNA polymorphism and copy number between high-altitude populations and low-altitude counterparts for each species. These mitochondrial gene sequence and content observations consistently indicate that birds have distinctly different mitochondrial acclimation processes to cope with high-altitude hypoxia than mammals. Our study provides insight to the general and specific characteristics of mitochondrial phenotypic acclimatization and genetic adaptation to high-altitude hypoxia in birds and mammals.

## Supplementary Material

Supplemental Material
