# Supplementary material for: Analysis of mitochondrial DNA sequence and copy number variation across five high-altitude species and their low-altitude relatives
Source: Mitochondrial DNA B Resour. 2018 Aug 27;3(2):847–51. doi: 10.1080/23802359.2018.1501285 (PMC7799994; doi:10.1080/23802359.2018.1501285)
Supplement: Supplemental Material [file TMDN_A_1501285_SM4097.docx]

**SUPPLEMENTARY MATERIAL**

Primer sequences used in “**Analysis of mitochondrial DNA sequence and copy number variation across five high-altitude species and their low-altitude relatives**” Rui Liu, Long Jin, Keren Long, Qianzi Tang, Jideng Ma, Xun Wang, Li Zhu, An’an Jiang, Guoqing Tang, Yanzhi Jiang, Xuewei Li, and Mingzhou Li

**Table S1**. Primer sequences used for mtDNA sequencing in five species.

| **Primer ID** | **Primer sequence (5' to 3')** | **Primer ID** | **Primer sequence (5' to 3')** | **Amplicon**  **size (bp)** |
| --- | --- | --- | --- | --- |
| **Chicken** | | | | |
| c-1-f | ACCTGCGTTGCGTCCTAT | c-1-r | TTCTAAGGGCGGGTTTCA | 1355 |
| c-2-f | AACTACGAGCACAAACGC | c-2-r | TTGGGAAACAGTCGGGTC | 1406 |
| c-3-f | AAACCAAAGACCCGACTG | c-3-r | ATAGATAGAAACCGACCTG | 648 |
| c-4-f | TTGTATGAATGGCTAAACG | c-4-r | AGGAATAGGACGGTGGTT | 1471 |
| c-5-f | TCCTATTCCTGAACCCAAGC | c-5-r | CTTGTTTGGTTAGTTCTTGGAT | 1291 |
| c-6-f | GAGCATAACCAACGCCTGAT | c-6-r | TTAGGTAAAGAGTGCCAATGTC | 1249 |
| c-7-f | TTCTTCTACCTCCGACTT | c-7-r | GTTGGTATAGGATTGGGT | 1234 |
| c-8-f | TCCTTGGCTTCATTGTAT | c-8-r | CAACTTCTTGGGCATCTA | 1023 |
| c-9-f | AACACCGTAGATGCCCAAGA | c-9-r | CCGTTAGGGCGGAGATTG | 1333 |
| c-10-f | TCAATCTCCGCCCTAACG | c-10-r | GGCTGCTGCTTCAAATCC | 639 |
| c-11-f | TTCTTCGTCGCTACAGGG | c-11-r | GGTGAGTTTGATAATGGGT | 1522 |
| c-12-f | ACTATCATCGCCCTCCTC | c-12-r | GCTATCAGGTTGGTGGTT | 1561 |
| c-13-f | ACGGACTACACCTATGACTG | c-13-r | ATAAATGGTTCGGATGCT | 1301 |
| c-14-f | CTAATCCCAACAACAATCT | c-14-r | ATGTGGCAAGTAGTGTAAGT | 1105 |
| c-15-f | CACCTCCTGCCTAACCAT | c-15-r | GTCCGATGTGAAGGAAGATA | 1017 |
| c-16-f | TTTCGCCCTCACAATCCTTACAACG | c-16-r | CGTGGGTTGTCTCGGGGGGCAATTC | 1377 |
| c-17-f | CCCACAATCGGAACACTA | c-17-r | ACGCAAACCGTCTCATCG | 1570 |
| **Pig** | | | | |
| p-1-f | GAGAATGCCCTCCAGATC | p-1-r | GGTTCAAAGTACCCATAT | 963 |
| p-2-f | AATACCCACCATACGAAA | p-2-r | CCTTTGCACGGTCAGAAT | 1219 |
| p-3-f | GAGTAACAAGAAGCCTTT | p-3-r | GGGTATTGGTAGTGGAAC | 1282 |
| p-4-f | CTCACCCTAGTAGAACGA | p-4-r | GGTAGTGTAGATAATGGGATTT | 1132 |
| p-5-f | AGGTTCAAACCCTCTTAT | p-5-r | AGGAGTAGGCTAGTCGTA | 1062 |
| p-6-f | TATCCCATACATGAAACAAG | p-6-r | CAAGGTGTAGGGAGAAAA | 1193 |
| p-7-f | GACTCGTACCGCTAATA | p-7-r | GACATCCGTGTAGTCATT | 1254 |
| p-8-f | TAGGCTCATTCATCTCAC | p-8-r | TCTGGGCTTGCTGGGTAT | 1195 |
| p-9-f | AACCAAGCATGAGCAAAA | p-9-r | GCATACCATTGAGGGGAG | 1237 |
| p-10-f | ATCCCAGGACGACTAAAC | p-10-r | CTGATAGGGCTCCGGTAA | 1145 |
| p-11-f | ACAGCCAACATTACAGCA | p-11-r | CGATTAGTACGAGTAGGGA | 1114 |
| p-12-f | TAATCATCGGATCTACTTTC | p-12-r | TCGGGTTGTGGTTTCTTT | 1210 |
| p-13-f | TCTCCGACTCACTATCAG | p-13-r | TAATGTGGTGGGTGTATT | 1101 |
| p-14-f | CCCCATCCATCAATCTAA | p-14-r | GTAATGCTGATACGGGAG | 1207 |
| p-15-f | ATCGGATGATGACACGG | p-15-r | ATGTTGGTAGGCGGTGT | 1153 |
| p-16-f | AACAGCCCTAATCGTAAC | p-16-r | CCTAAGAGGGAACCGAAG | 1055 |
| p-17-f | TCCACCACTTACAATCAA | p-17-r | ATGGGTGTTCTACGGGTT | 1226 |
| p-18-f | TATTCGCCTACGCTATCC | p-18-r | GCTGAGTCCAAGCATCCC | 1032 |
| p-19-f | CGCGTGAAACCAGCAACC | p-19-r | TGGCACGAGATTTACCAACT | 1029 |
| **Cattle** | | | | |
| ca-1-f | ACTGTGCTGTCATACATTT | ca-1-r | CATAGTGGGGTATCTAATC | 836 |
| ca-2-f | GGGAAACAGCAGTGACAA | ca-2-r | TCCCTTGCGGTACTTTCT | 994 |
| ca-3-f | GACAAGTCGTAACAAGGTA | ca-3-r | CAATGAGCGATAGAGTGA | 1353 |
| ca-4-f | GAGAAGACCCTATGGAGC | ca-4-r | TATGAAGAAGAGGGCAAA | 1262 |
| ca-5-f | TAGCAGAAACAAACCGAG | ca-5-r | CTTCTGTAGCTCGTGGGT | 760 |
| ca-6-f | ACCTACTCCTAAGAATCCAA | ca-6-r | GAAGGCTCTTGGTCTGTT | 1198 |
| ca-7-f | ACCAAAATGAATAATCATCC | ca-7-r | AGTTGATGGCTCCTAAAA | 1129 |
| ca-8-f | AGGAACAGGCTGAACCGT | ca-8-r | ATGCTTCTCAGATGATGAAAAC | 1061 |
| ca-9-f | GATACATGAGCCAAAATC | ca-9-r | GTAAAGAAGGAAGAGCAA | 713 |
| ca-10-f | TAAAATAAGAAAGGAAGG | ca-10-r | CATGTTGACGTGTCTAGT | 931 |
| ca-11-f | GCACTAACCTTTTAAGTT | ca-11-r | GTGTTGTCATGCAGATAT | 889 |
| ca-12-f | ACTTGCCCATTTCTTACC | ca-12-r | ACCGAAACTAGCTGATTG | 1144 |
| ca-13-f | CTATTACCTGAGCCCATC | ca-13-r | GGGTATAAGTATAATTGTTGGA | 1171 |
| ca-14-f | CTTACCATAGCCCTCTTC | ca-14-r | GTGATTTTAGGTCCGTTT | 1298 |
| ca-15-f | CGGACTCTATTTCCTATT | ca-15-r | ACTTTTATTTGGAGTTGC | 1145 |
| ca-16-f | CAACAATCAACTTAATTGGAGA | ca-16-r | GTAGTGCTGAGACGGGAG | 1208 |
| ca-17-f | AGCACTATTCGTCACATG | ca-17-r | AGAAGGGAGGATGCTGAT | 1383 |
| ca-18-f | CTTATACCAACGCCTGAG | ca-18-r | TTGGTTATACAACGGCTA | 926 |
| ca-19-f | AAATCAGCATCCTCCCTT | ca-19-r | ATTTGTGCCGATGTATGG | 1244 |
| ca-20-f | TATCTGCCGAGACGTGAA | ca-20-r | ATGGGTGTTCGACTGGTTG | 836 |
| ca-21-f | CTCCACGAAACAGGCTCC | ca-21-r | TGGCCCTGAAGAAAGAAC | 1178 |
| ca-22-f | TACCATGCCGCGTGAAAC | ca-22-r | TGCTGGTGCTCAAGATGC | 332 |
| **Goat** | | | | |
| g-1-f | TAAAGCAAGGCACTGAAA | g-1-r | TAAATCCTCCTTTGGTCAT | 834 |
| g-2-f | ATACCGCCATCTTCAGCA | g-2-r | CGTTAGGCATGTCACCTCT | 758 |
| g-3-f | CCGTAAGGGAATGATGAA | g-3-r | CAATACTGGAAATGCTGGA | 756 |
| g-4-f | GATTAAAAGAAGTAAAAGGAACTCG | g-4-r | GCTACGGCTAGAAGAATGG | 911 |
| g-5-f | CCGCTATCAAAGGTTCGT | g-5-r | GGTCAGGTCAAATGGTGC | 892 |
| g-6-f | CGAGCAGTAGCACAAACA | g-6-r | CAGATGAGTAGTCAGTGGGAG | 855 |
| g-7-f | GTCACTACCCATTCTCCTATC | g-7-r | TTTGGTTTAGTCCTCCTCA | 768 |
| g-8-f | ATAACCCACGAGCCACAG | g-8-r | CAGGAATTAAGTAAATTATACTTGC | 967 |
| g-9-f | AACAGCATTATCTTACCCACC | g-9-r | GTCAGTTGCCAAACCCTC | 847 |
| g-10-f | CAAAGACATCGGCACCCT | g-10-r | CTCATACAATAAATCCTAGAAACCC | 830 |
| g-11-f | CAGGAGCCTCAGTAGACC | g-11-r | AGGAAATGTTGTGGGAAG | 877 |
| g-12-f | CTTCTTCCCACAACATTTC | g-12-r | CAACTCGGTTATCTACCTCTA | 834 |
| g-13-f | CACCAGCACCATAGACGC | g-13-r | CCAATAAATAGGATTAGGGACA | 997 |
| g-14-f | CCCCTTGTTACCCTTATT | g-14-r | GTTACGGTCTCCTTCCAT | 1100 |
| g-15-f | AACATCACAGCAGGACACT | g-15-r | CGGATTATACCGAAACTAAC | 1037 |
| g-16-f | GACGGAGTCTACGGTTCA | g-16-r | GGCATAGGAGGGAGGATA | 799 |
| g-17-f | TAGCCATCACATTTCTCCT | g-17-r | GACGCTCTGTTTGGTTTC | 961 |
| g-18-f | TAACATTCACCGCTACAGA | g-18-r | TTTGGGTTTAGGGATAGAA | 1033 |
| g-19-f | TACCCATCACATCAACAACA | g-19-r | CCTTCTCAGCCGATAAATAG | 761 |
| g-20-f | CTATTTATCGGCTGAGAAGG | g-20-r | TAATGAGGGCTGTTGTGG | 706 |
| g-21-f | ACCACAACAGCCCTCATT | g-21-r | GGGATTCTGGGTTCTTCA | 837 |
| g-22-f | CCCCTGTATCATAAATAACCCAATC | g-22-r | TGGTGTAGTAAGGGTGAAAT | 1143 |
| g-23-f | GGGATTCTCAGTAGACAAAGC | g-23-r | CTAGTGGACGGGATACGC | 1420 |
| g-24-f | GCCTTCCACTTTATCCTC | g-24-r | GGTTGTTGGCATGTCTGTA | 873 |
| g-25-f | AACTATTCCCTGAACCACTA | g-25-r | GTCCAGCTACAATTTATGCT | 972 |
| g-26-f | TTATCAGACATCTGGTTCTT | g-26-r | ACCAACCCTGGTCAACATGGCTTAG | 768 |
| **Sheep** | | | | |
| s-1-f | GTTTGGTCCCAGCCTTCC | s-1-r | CCCATTTCTTCCCACTCC | 711 |
| s-2-f | GGTGCTTTATACCCTTCT | s-2-r | TAGGCATGTCACCTCTAC | 840 |
| s-3-f | GCAAGGGAATGATGAAAG | s-3-r | GATGCTGGAGGTGATGTT | 742 |
| s-4-f | AAATGGGTACAACCTTCA | s-4-r | ATAGAAACCGACCTGGAT | 983 |
| s-5-f | TGACCTCGGAGAACAGAA | s-5-r | TAGGGTTAGTGCTAGGATGG | 731 |
| s-6-f | TCCCAGTACGAAAGGACA | s-6-r | TAGCGATTTGATGGTGAA | 983 |
| s-7-f | CCTTTACCCTTTCTACAC | s-7-r | TGTTATTCAGCCTATGTG | 1263 |
| s-8-f | AACCAAACCCAACTACGA | s-8-r | ATGGCTGAGTAAAGCAATAGAC | 905 |
| s-9-f | TTCAAAGCCCTAAGCAAG | s-9-r | AAGTAAGCCCGTGTATCG | 837 |
| s-10-f | TAACGGACCGAAACCTGA | s-10-r | TGCGTCTTGAAAGCCTAG | 1095 |
| s-11-f | TAGAATGACTAAACGGATGT | s-11-r | CAATGAGGGTAACGAGGG | 1200 |
| s-12-f | CCCTCGTTACCCTCATTG | s-12-r | AACCGTAAACTCCGTCTG | 1211 |
| s-13-f | TCAGACGGAGTTTACGGT | s-13-r | TGTGGCAGTGAATGTTAT | 1334 |
| s-14-f | GCTAATCAGCCTCACAAG | s-14-r | GAGAATGGCAACAATGAC | 811 |
| s-15-f | CAAAGCCCATGTAGAAGC | s-15-r | TTCGGTAAATAAGAAGGTGA | 800 |
| s-16-f | TAATGGCTGCCTGATGAC | s-16-r | GTAGTGCTGAGACGGGTG | 1258 |
| s-17-f | ATTGGCTGAGAAGGAGTT | s-17-r | AATGCGAGTGCTGTAGAT | 855 |
| s-18-f | ATTGAATCCGCTAATACG | s-18-r | GTGGGTTACAGAGGAGAA | 1424 |
| s-19-f | CAATACACTATACACCTGACA | s-19-r | GTTGGTTGTTCTCCTTCT | 1047 |
| s-20-f | GAGTCCTCGCCCTAATCC | s-20-r | GCTGAGTCCAAGCATCCC | 1299 |
| s-21-f | GGCATCTGGTTCTTTCTT | s-21-r | TGCTTGATACCTGCTCCT | 640 |

**Table S2**. Primer sequences used to determine mtDNA copy number in five species.

| **Gene** | **Primer sequence (5’ to 3’)** | **Gene** | **Primer sequence (5’ to 3’)** |
| --- | --- | --- | --- |
| **Chicken** | | **Goat** | |
| *ATP6* | F:CCTCCCATCACTCCTTCT | *ATP6* | F:TAACAACCGCCTCATCT |
|  | R:GTGACCTGCCTTGTTTAG |  | R:CCTGCTCATAAGGGAAT |
| *COX1* | F:ACAAAGACATTGGCACTC | *COX1* | F:CAAAGACATCGGCACCC |
|  | R:AGAAGATTATGACGAAAGC |  | R:GCCAAACCCTCCAATCA |
| *ND1* | F:CTTACGAGCAGTAGCAC | *ND1* | F:TACGAGCAGTAGCACAA |
|  | R:CCTTCGGTCAGGTCAAA |  | R:CCTTCGGTCAGGTCAAA |
| *AGRT1* | F:TGGCCATAGTGCATCCAGTG | *CSN2β* | F:TGGAAGAAGGCCTCCTATTGTC |
|  | R:ACGATGAATGATGACGGGCA |  | R:AGGTTCCCGGAATCCTACTTG |
| **Pig** | | **Sheep** | |
| *ATP6* | F:TATTTGCCTCTTTCATTGCCC | *ATP6* | F:ATTACAGGCTTCCGCAACA |
|  | R:GGATCGAGATTGTGCGGTTAT |  | R:TACGGCAAGGGCTACTGG |
| *COX1* | F:ACTACTGACAGACCGCAACC | *COX1* | F:GTGGATTCGGCAACTGAC |
|  | R:TCCAATGGACATTATGGCTC |  | R:TCAACCTGTTCCTGCTCC |
| *ND1* | F:GCCACATCCTCAATCTCCAT | *ND1* | F:CCGCCACATCCTCAATCTCA |
|  | R:GATTAGAGGGTAGGGTATTGGTAG |  | R:ACGGCTAGGCTTGATATGGC |
| *GCG* | F:GAATCAACACCATCGGTCAAAT | *SCD* | F:TTGCTTGACTGCTTGTCCCA |
|  | R:CTCCACCCATAGAATGCCCAGT |  | R:CTCCCTGAGCCGTTGTTCTT |
| **Cattle** | |  |  |
| *ATP6* | F:TTACCCCATTCATTCACA |  |  |
|  | R:GCGAGGGCTATAGGTTGA |  |  |
| *COX1* | F:CCTCTATAGTTGAAGCTG |  |  |
|  | R:TTGTAATGAAGTTGATGG |  |  |
| *ND1* | F:CTACGAGCAGTAGCACAA |  |  |
|  | R:TATGAAGAAGAGGGCAAA |  |  |
| *AGRT1* | F:TGCCAGCGTGTTTCTACT |  |  |
|  | R:AAGCCTTCTTGAGGGTCT |  |  |
